# Immunological Tumor Microenvironment of Solitary Fibrous Tumors—Associating Immune Infiltrate with Variables of Prognostic Significance

**DOI:** 10.3390/cancers16183222

**Published:** 2024-09-21

**Authors:** Emilio Medina-Ceballos, Isidro Machado, Francisco Giner, Álvaro Blázquez-Bujeda, Mónica Espino, Samuel Navarro, Antonio Llombart-Bosch

**Affiliations:** 1 Pathology Department, Hospital Clínico Universitario de Valencia, 46010 Valencia, Spain; emilio.medinacs@gmail.com (E.M.-C.);; 2 Pathology Department, Instituto Valenciano de Oncología, 46009 Valencia, Spain; 3 Patologika Laboratory, Quirón-Salud, 46010 Valencia, Spain; 4 Cancer CIBER (CIBERONC), 28029 Madrid, Spain; 5 Pathology Department, University Hospital La Fe, 46010 Valencia, Spain; 6 Pathology Department, University of Valencia, 46010 Valencia, Spain; 7 School of Medicine, University of Valencia, 46010 Valencia, Spain

**Keywords:** solitary fibrous tumor, microenvironment, prognosis, TILS, immunological response

## Abstract

**Simple Summary:**

Solitary fibrous tumors (SFTs) are tumors that can vary greatly in how they behave, with some being harmless while others become aggressive and life-threatening. This study evaluated 52 fusion-confirmed SFTs with clinical follow-up. The aim was to analyze the immune intratumoral infiltrate and correlate the presence of specific immune cells and risk stratification systems to see if the presence or absence of certain cells could help predict how the tumor will behave or correlate with other clinicopathologic characteristics. The research found that, while certain immune cells were associated with specific tumor features with prognostic significance, these immune cells did not reliably predict the tumor’s overall risk level. This suggests that the immune environment within SFTs is complex and may not be useful on its own for determining the tumor progression risk. Nonetheless, the presented results give an insight into the relationship between immune tumor cells and histologic characteristics such as necrosis, proliferation index, and neoplastic cell morphology.

**Abstract:**

Background and objectives: Solitary fibrous tumors (SFTs) are morphologically heterogeneous tumors characterized by the *NAB2::STAT6* gene fusion. Clinical outcomes may vary widely, and while most cases have favorable outcomes, some can progress to aggressive disease, manifesting as recurrence and metastasis, and ultimately resulting in patient death. Herein, we analyze the immunological tumor microenvironment (ITME) of SFTs, aiming to determine its prognostic value and correlation with established risk stratification systems (RSSs). Methods: A retrospective observational multicenter study of 52 fusion-confirmed SFTs with clinical follow-up data. Immunohistochemical analysis including CD163, CD68, CD3, CD8, CD20, PDL-1, PD-1, and LAG1 were evaluated in tissue microarrays, using an analog scale with scores ranging from 0 to 3 (0 = ≤9, 1 = 10–49, 2 = 50–99, and 3 = >100 positive cells per 10 high-power fields). The expression of these markers was correlated with clinical outcomes, morphological characteristics previously evaluated in whole slide tissue sections (hypercellularity/hypocellularity, round–oval or spindle dominant constituent cell (DCC) morphology, and necrosis), Ki67, overall survival, and RSS. Results: Only one of the fifty-two cases studied showed progression. In the multivariate analysis, neither the presence nor absence of immune cells (B-lymphocytes, T-lymphocytes, and macrophages) showed any association with the assessed RSSs (Demicco, Sugita, G-score, and Huang). Interestingly, the case that showed progression had high immune infiltrate with expression of CD68, CD163, CD8, and CD20 markers (score of 3). Round–oval cell morphology was associated with the presence of higher levels of CD163 macrophages. Lastly, the scant presence of CD20+ lymphocytes correlated with less necrosis, and cases with higher PDL-1 expression correlated with increased Ki67 values. All cases were negative for LAG-1 and PD-1. Conclusions: SFT ITME components correlated with independent variables with prognostic significance. Nevertheless, ITME did not correlate with RSS scores.

## 1. Introduction

Solitary fibrous tumors (SFTs) are rare fibroblastic tumors, formerly known as hemangiopericytoma. The reported incidence rate is up to 6.1 cases per 10,000,000 people per year, representing 3.7% of soft tissue sarcomas (STSs) [1,2]. First described by Klemperer et al. in 1931 [3], SFT was initially thought to occur only in the pleura and lung, but is now recognized to occur in virtually any anatomic location. Known as the “great simulator” of soft-tissue neoplasms due to its many morphological appearances and differential diagnoses, accurate diagnosis relies on a combination of clinical, pathological, immunohistochemical, and molecular features. The *NAB2::STAT6* gene fusion, identifiable via immunohistochemistry or molecular biology techniques, serves as a specific cytogenetic hallmark and is essential for the diagnosis [3]. Clinical outcomes of SFT vary widely; while most cases exhibit a favorable and indolent course, a significant minority can progress to aggressive disease manifesting as recurrence and metastasis, potentially leading to patient death [4]. This variability in clinical outcomes requires the development of systems to stratify risk of recurrences or metastasis in SFTs [5]. The most widely used and accepted risk stratification system (RSS) is that proposed by Demicco et al. [6]. Initially published in 2012, Demicco’s system classifies SFTs into three risk categories (low, medium, and high risk), based on age, tumor size, and mitotic count [6]. This system has been validated in several series, demonstrating its clinical utility and reproducibility [7,8]. However, Demicco’s system has its limitations, thus promoting the development of new RSSs incorporating additional variables. The Sugita RSS includes the Ki67 index [9]. The G-score accounts for gender, necrosis, and mitotic count [10], while the Huang RSS introduces the dominant constituent cell (DCC) morphology, distinguishing between round or spindle cell types [11].

The prognostic significance of the immunological tumor microenvironment (ITM) in SFTs remains poorly studied. This study aims to analyze the ITME of SFTs to determine its prognostic value and correlation with established RSSs. By examining the presence and impact of immune cells such as B-lymphocytes, T-lymphocytes, and macrophages, this study also seeks to elucidate the potential role of the ITME in SFT progression and patient outcomes.

## 2. Materials and Methods

The study included 52 fusion-confirmed cases of SFTs, all demonstrating positive nuclear staining for STAT6 by immunohistochemistry, and following our previously published series evaluating RSSs for SFTs alongside histological, immunohistochemical, and molecular findings [8,12,13]. The study was conducted in accordance with the principles of the Declaration of Helsinki and approved by the local Ethics Committee. Formalin-fixed, paraffin-embedded tissue samples were retrieved from the Pathology Departments of the Clinical Hospital University of Valencia, University Hospital “La Fe”, and Instituto Valenciano de Oncología (IVO). Clinical data, including age, gender, tumor site, location, size, depth, treatment (surgery, chemotherapy, radiotherapy), and follow-up data (recurrence, metastases, and final outcome), were also collected from the corresponding files. Patients were followed up for a median period of 64.7 months (range, 17–128 months), providing robust longitudinal data. Additional pathological factors associated with aggressive behavior, such as DCC, ≥4 mitoses/10 HPF, cytological atypia/pleomorphism, tumor necrosis, infiltrative margins, and/or tumor size ≥10 cm, had been assessed previously in whole slide tissue sections to determine their impact on prognosis and overall survival. All cases were classified using four different RSSs (Demicco, Sugita, G-score, and Huang).

### 2.1. Immunohistochemistry

Immunohistochemistry staining was carried out on 3–4 μm thick formalin-fixed paraffin-embedded tissue microarrays. The markers studied included CD163 (10D6—Biocare, Concord, CA, USA), CD68 (KP1—Dako, Santa Clara, CA, USA), CD3 (Polyclonal Rabbit—Dako), CD8 (C8/144B—Dako), CD20 (L26—Dako), PDL-1 (PA5-28115—Thermo Fisher Scientific, Waltham, MA, USA), PD-1 (SP142—Roche, Basel, Switzerland), and LAG1 (Gennova Scientific, Sevilla, Spain). The extent of positive immunohistochemical reaction was scored ranging from 0 to 3 (0 = ≤9, 1 = 10–49, 2 = 50–99, and 3 = >100 positive cells per 10 high-power fields) for all the antibodies, with the exception of PDL-1, which was graded following the same scoring method as used in the study of Kamamoto D., which used a grading from 0 to 2 according to the positive cell ratio (0% negative = 0, 1–50% low expression = 1, and >50% positive tumor cells high expression = 2) [14].

All sections were evaluated independently and read in a blind manner by three pathologists (EM-C, IM, and ALLB). Discordant cases were evaluated using a multiheaded microscope to achieve consensus. Standard positive and negative controls were used throughout. The scores of all observers were recorded, and in cases of disagreement the score was determined by consensus.

### 2.2. Statistical Analysis

The statistical analysis was conducted using Python (3.8.5), primarily using the following libraries: NumPy (1.19.2), Pandas (1.2.3), SciPy.stats (1.6.2), and Seaborn (0.13.2). This approach provided flexibility and customization in data management, analysis, and visualization. For all analyses, *p* < 0.05 was considered statistically significant. The overall follow-up period and the interval until an event (recurrence or metastasis) were calculated starting from the date of surgical removal or biopsy. Follow-up for patients who did not experience recurrence was considered up to the date of their most recent clinical check-up or last radiological control.

## 3. Results

**Figure 1:**
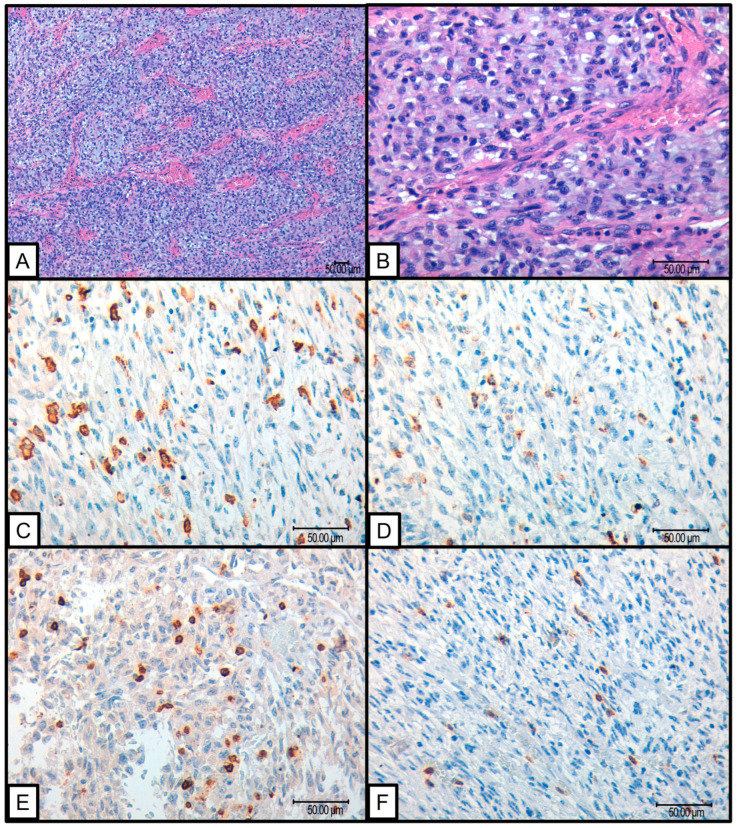
SFT that presented progression: (**A**,**B**) H&E 10× and 40× SFT with round to oval dominant constituent cell morphology; (**C**) 40× CD163 positive macrophagic infiltrate (**D**) 40× CD 68 positive macrophagic infiltrate; (**E**) 40× CD3 positive intratumoral lymphocytes; (**F**) 40× CD8 positive intratumoral lymphocytes.

Several findings emerged from the statistical analysis of the cohort and their ITME. Fifty-two cases of histologically confirmed SFTs were studied, of which one showed tumor progression. This particular case involved a pulmonary SFT measuring 5 cm in a 70-year-old patient. Microscopically, the tumor exhibited a round–oval morphology with 1 mitosis per 10 high-power fields, no necrosis, a Ki67 index of 25%, and a Demicco risk stratification score of 3 (intermediate risk). The tumor recurred 72 months after the initial diagnosis. In this case, the tumor demonstrated a significant immune response with high levels of CD163 (3+), CD68 (3+), PDL-1 (2+), CD3 (3+), and CD8 (3+) infiltrating cells (Figure 1).

No association was found between ITME markers (CD163, CD68, CD3, CD8, CD20, PDL-1, PD-1, and LAG1) and the risk categories of the different RSSs. Contingency tables and chi-squared tests indicated that the *p*-values were substantially higher than 0.05, suggesting no significant correlation between these markers and the risk scores (Supplementary Figure S1). Furthermore, an analysis of the absolute percentage differences between observed and expected frequencies confirmed minimal discrepancies, reinforcing the lack of association.

All cases were negative for PD-1 and LAG1 expression, showing no variability for these markers among the studied samples.

**Figure 2:**
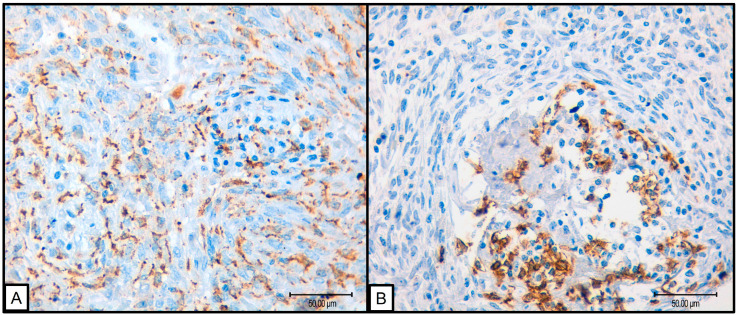
(**A**) 40×, SFT with round cell morphology presenting abundant macrophagic CD163 positive infiltrate; (**B**) 40×, SFT presenting B cell CD20 positive infiltrate.

Due to the limited number of progression events (*n* = 1), survival analysis using Kaplan–Meier curves and log-rank tests was not feasible. Therefore, prognostic impact was assessed through comparisons with various clinical and pathological variables. Notably, significant associations were observed between low levels of CD68, CD8, and CD20 markers and reduced progression. However, given that progression was seen in only one case, these results should be interpreted with prudence. Additionally, high levels of CD163 were associated with round–oval cellular morphology (*p* = 0.047), while low CD20 levels correlated with less necrosis (*p* = 0.013) (Figure 2).

For continuous variables (age, size, Ki67 index, and mitotic count per 10 high-power fields), box plots and ANOVA tests were employed (Supplementary Figure S2). The most notable finding was the significant increase in Ki67 levels in tumors with higher PDL-1 expression. Other significant associations, such as age with CD8 infiltrate and size with CD20 infiltrate, were detected. Nevertheless, these associations did not present consistent patterns, suggesting that the results might be due to random chance given the high number of comparisons made.

## 4. Discussion

In the last decade, there has been a surge in the study of and interest in the tumor microenvironment (TME). This trend has been driven by the development of new immunotherapeutic treatments that have shown promising results in managing various types of malignant tumors [15,16,17]. Nevertheless, the study of the ITME dates back to the second half of the 19th century when R. Virchow proposed a relationship between inflammation and cancer [18]. One century later, Dvorak demonstrated that carcinogenesis and inflammatory conditions share common developmental pathways. Both tissues are composed of similar stromal cell types, such as infiltrated inflammatory cells and angiogenesis-related cells; the only difference he pointed out was that while wounds eventually healed, tumor tissues did not heal [19].

The TME is a highly complex and dynamic system, comprising various cellular and non-cellular components that surround tumor cells [20]. Three main actors are involved in the TME: first, vasculature, which is indispensable for tumor growth, fulfilling the neoplastic cells’ high metabolic requirements, including tumor-associated endothelial cells, which favor migration, and proliferation via multiple growth-signaling pathways [21]; second, cancer-associated fibroblasts that modulate tumor stroma, facilitating the synthesis of extracellular matrix and secretion of metalloproteinases, generating a remodeling of the cellular scaffold [22]; and third, the immune cells, both innate and adaptive, that interact with the neoplastic cells, influencing tumor behavior and response. All these factors orchestrate complex interrelations via cytokines, growth factors, and hormones resulting in tumor growth and migration, and providing opportunities for research into novel therapeutics [20,23,24].

Immune infiltrate serves a crucial role in neoplasms, being either pro-tumorigenic or anti-tumorigenic. At this time, knowledge of the influence of immune infiltrate in STS is still limited compared to more extensive research being conducted in carcinomas [25]. Multiple studies have been conducted to evaluate the role of ITME in the treatment and prognosis of STS. A study conducted by R. Issels evaluated 109 patients and found that high tumor-infiltrating lymphocyte (TIL) counts, which increase after neoadjuvant chemotherapy, are predictive of improved local progression-free and disease-free survival in STS, with significant post-treatment changes in T cell subsets [26]. A broader study by Sorbye S. et al., which included different sarcoma types, highlighted that high TIL density, particularly of CD20 lymphocytes, following neoadjuvant therapy, correlates with better prognosis in terms of local progression-free and disease-free survival, indicating the potential of TILs as a predictive biomarker for therapeutic response and survival outcomes in sarcoma patients [27]. In a series conducted at the Memorial Sloan Kettering Cancer Center, fifty sarcoma patients, including two SFTs, were studied to evaluate the prevalence and implications of TILs and PDL-1. The authors found that TILs and PDL-1 positive macrophages and lymphocytes were common, and that low levels of CD3+ and CD4+ lymphocytes correlated with better overall survival. However, PD-L1 expression in tumor cells was rare and did not significantly correlate with clinical outcomes. The authors also noted some limitations in the study and discrepancies with other results published in the literature [28].

There are few studies with large cohorts focusing on ITME in specific types of STSs and they often present limitations due to the low incidence of these tumors. Our study was not exempt from these challenges, as our main limitation was that only one out of the fifty-two cases showed progression. However, this did not prevent us from conducting a valid statistical analysis, since our cohort provided a comprehensive sample of different RSS scores and nearly all of the many different morphologies described in SFT. This representation of multiple tumor morphologies highlighted the varied nature of this tumor entity. The wide range of histological presentations, from benign paucicellular appearances with bland cytology, to lesions with epithelioid or small round cell morphologies, provided us with a broad sample of histological variables of prognostic significance, such as necrosis, proliferation indexes, and cell morphology, which allowed us to achieve our primary objective of evaluating possible correlations between the ITME, RSS scores, and variables of prognostic significance.

Our findings regarding TILs demonstrate that the presence of CD3, CD8, and CD20 lymphocytes does not correlate with the RSS categories. Nevertheless, the presence of CD20 lymphocytes was found to be associated with the presence of necrosis. Necrosis represents an important histological feature, and is considered in almost all RSSs due to its consistent correlation in several multivariate analyses [6,7,8,9,10,11,12]. This B cell infiltrate may arise when tumor cells enter into apoptosis resulting in necrosis, and damage-associated molecular patterns (DAMPs) are released from damaged cells, signaling tissue damage to the immune system and inducing an inflammatory response. DAMPs come from various cellular compartments (cytosol, mitochondria, nucleus, plasma membrane, endoplasmic reticulum) and activate immune cells, promoting an adaptive immune response and inflammation [29,30]. While DAMPs can activate and orchestrate immune responses against tumors, they can also contribute to tumor invasion, growth, and spread by promoting neoplastic angiogenesis and matrix dissolution via matrix metalloproteinases. This dual role of DAMPs underscores a complex balance between tumorigenesis and the immune response, where they both combat and facilitate tumor progression [31]. The presence of B cell lymphocytes in various epithelial tumors, such as lung, ovary, and prostate adenocarcinomas, and also melanoma, has been correlated with better survival rates [32,33,34,35]. In the above-mentioned article by Sorbye S. et al., the authors concluded that a high density of CD20+ TILs in STS acts as a positive prognostic indicator in tumors with wide resection margins [27]. Nonetheless, in a subsequent publication, the same did not hold true for patients with non-wide resection margins. Furthermore, it was suggested that a high density of CD20+ lymphocytes in the peritumoral capsule is a negative prognostic indicator, regardless of resection margins [36]. A large study conducted in the Centre de Recherche des Cordeliers, France, classified STS into five groups based on the TME, finding that sarcomas classified as “high in B cell infiltrate and presence of tertiary lymphoid structures” correlated with improved survival and higher rates of response to anti-PD1 therapy [37].

Regarding CD4 and CD8-positive TILs, our study did not find any relevant associations with direct or indirect factors that affect tumor progression. This finding concurs with the results published by Zhang R. et al., where no association was found between these markers and progression-free survival in a large series of 406 SFT cases [38]. A paper published by Smolle M. et al. aimed to study the presence of T-lymphocytes and immune check point markers in STSs of the extremities and trunk. Notably, they found that higher levels of CD3+ T cells were observed in G3 STS compared to G1 or G2, indicating a potential link between immune infiltration and tumor aggressiveness. In older patients, there was a significant increase in CD3+ CD4+ helper T cells, PD1+ CD3+ T cells, CD3+ CD4+ FOXP3+ regulatory T cells, and PD1+ CD3+ CD8+ cytotoxic T cells compared to younger patients, highlighting age-related differences in immune profiles. Histological subtype analysis revealed that myxofibrosarcoma exhibited higher TIL levels than synovial sarcoma and leiomyosarcoma, with undifferentiated pleomorphic sarcoma also showing elevated levels of specific TILs compared to other subtypes. Importantly, high regulatory T cell levels were associated with increased risk of local recurrence, while large tumor size and G3 grading correlated with a higher risk of distant metastasis. Furthermore, advanced age, high CD3− PD-L1+ levels, and low FOXP3+ regulatory T cell levels were linked to worse overall survival [39]. The results are similar to those reported by the Memorial Sloan Kettering Cancer Center group previously referenced [28]. This sarcoma TME study did not include SFTs in the series, which may explain the discrepancy between their results and the absence of correlations of CD4+ and CD8+ TILs with RSS, as well as variables with prognostic significance and age in our series.

Tumor-associated macrophages are a major component of the immune infiltrate, and are present to a varying extent in all neoplastic proliferations [40] and play an important role in the TME, supporting neoplastic progression and resistance to treatment by providing malignant cells with trophic and nutritional support [41]. Tumor-associated macrophages can have a dual function in carcinogenesis, being supportive or inhibitory depending on the tissue and disease stage. Evidence indicates that macrophages, rather than being tumoricidal as classically suggested [42], in vivo adopt a pro-tumoral phenotype both in the primary and metastatic sites [43]. Macrophages also produce growth factors and cytokines that stimulate growth of neoplastic cells that have previously acquired cancer-associated mutations. Additionally, tumor-associated macrophages produce cytokines such as IL-1 that promote the accumulation of tumor cells at distant sites, providing a supportive environment for metastatic cells. Reactive oxygen and nitrogen intermediates generated by tumor-associated macrophages contribute to genetic instability, a hallmark of cancer that limits chemotherapy and targeted therapy effectiveness. Furthermore, they promote angiogenesis, lymphangiogenesis, and tissue remodeling by stimulating the deposition of fibrous tissue, all of which support tumor development and progression [40,43]. Macrophages express classical and nonclassical type I mayor histocompatibility complex molecules (MHCs), and this is normally associated with the presentation of antigens to T cells. Besides expressing MHC molecules, macrophages also present ligands for the inhibitory receptors PD-1 and CTLA-4. These inhibitory ligands are typically upregulated in activated immune effector cells, including T cells, B cells, and NK T cells, functioning as a safety mechanism to control immune response intensity and resolve inflammation. When PD-1 and CTLA-4 are activated by their respective ligands, they directly inhibit T cell receptor and B cell receptor signaling [40,43,44]. The relative importance of distinct macrophage differentiation pathways varies among different tumors, resulting in heterogeneous tumor-associated macrophages phenotypes and functions.

Our results revealed that a high infiltrate of macrophages CD163+ showed an association with round cell morphology. Round cell morphology is an independent morphologic variable proven to be linked to disease progression in 41–76% of SFT cases [8,11]. In the current study, the tumor that presented progression had round–oval cell predominance with abundant CD68+ and CD163+ macrophages. In the Zhang R. et al. SFT series, the authors demonstrated that the high density of CD68+ and CD163+ macrophages correlated significantly with a more rapid progression [38].

Notably, in our series, the case that exhibited disease progression demonstrated high expression of PDL-1, aligning with the findings reported by Kamamoto et al. in a study that analyzed intracranial SFTs, which reported that PDL-1 expression was correlated with a decrease in CD8-positive TILs. In these cases, there was an increase in treatment failure, and the authors concluded that TILs and PDL-1 expression could be associated with extracranial metastases [14]. These findings are supported by our results, where we observed a correlation between PDL-1 expression and Ki67. The Ki67 index in SFT has been extensively studied, repeatedly demonstrating that an increase in the proliferative index is directly related to an increased risk of disease progression [8,9,10,11,12]. The reported expression and significance of PDL-1 and PD-1 in sarcomas remains controversial, mainly due to wide variations in the results reported in the literature, where multiple series show different results that range from high percentages of tumors expressing the markers to studies reporting negative results [28,45,46,47,48].

A systematic meta-analysis was published in 2020 by Wang F. et al. [49], in which the prognostic value of PDL-1 expression was analyzed in bone sarcomas and STSs, including 39 independent studies with a total of 3680 tumors. Results indicated that high PDL-1 expression significantly correlated with poor overall survival, metastasis-free survival, and event-free survival. Specifically, PDL-1 overexpression predicted poor overall survival in osteosarcoma and leiomyosarcoma. Additionally, elevated PDL-1 levels were associated with higher tumor metastasis rates, advanced tumor grades, and increased T-lymphocyte infiltration. This meta-analysis emphasized the heterogeneity of PDL-1 expression across different sarcoma subtypes (primarily osteosarcomas, leiomyosarcomas, angiosarcomas, Ewing sarcomas, and non-specified STSs), suggesting that this variability might explain the inconsistent therapeutic outcomes observed in clinical settings. Moreover, the correlation between high PD-L1 expression and adverse clinical features like higher tumor grade and increased metastasis underscores the potential of PDL-1 as a prognostic biomarker [50]. Even so, although these results are based on a heterogeneous population of tumor types and the number of SFT cases is underrepresented, the findings are congruent with the previously presented literature on the impact of PDL-1 expression in SFTs and our correlation of PD-L1 with high levels of Ki67. Likewise, it should be noted that there is no standardized system for the evaluation of PDL-1 in STSs as there is in other epithelial tumors; this is an obstacle for PD-L1 assessment and ensuring consistent and reliable prognostic evaluations across studies.

## 5. Conclusions

Since sarcomas account for less than 1% of all adult solid malignant cancers [50], conducting studies with large patient series is inherently challenging and susceptible to study population limitations. Despite this, our study provides valuable insights into the TME of SFTs, a largely understudied area. The primary limitation of this study is the progression of only one case out of fifty-two, limiting our ability to perform a more extensive statistical analysis. In additionally, ITME quantification was conducted with an analog scoring system, which, while guided by strict established criteria and multiple expert blind review, may introduce variability.

Further validation with larger cohorts and more objective digital tools will be crucial in substantiating these results. Although our study did not identify a significant correlation between ITME markers and RSSs, the exposed findings contribute to the current limited knowledge on the TME of soft tissue tumors. As immunotherapy continues to show promise in other tumor types, understanding the TME of STS remains important for future research.

## Data Availability

The original contributions presented in the study are included in the article/Supplementary Materials; further inquiries can be directed to the corresponding author.

## Supplementary Materials

The following supporting information can be downloaded at: https://www.mdpi.com/article/10.3390/cancers16183222/s1, Figure S1: Contingency tables and chi-squared tests for RSSs and ITME markers; Figure S2: Box plots and ANOVA tests for numerical variables (age, tumor size, Ki67 index and mitotic count) and ITME markers.
