# Supplementary material for: Immunological Tumor Microenvironment of Solitary Fibrous Tumors—Associating Immune Infiltrate with Variables of Prognostic Significance
Source: Cancers (Basel). 2024 Sep 21;16(18):3222. doi: 10.3390/cancers16183222 (PMC11430690; doi:10.3390/cancers16183222)
Supplement: Supplementary file 1 [file cancers-16-03222-s001.zip › SUP 1.pdf]

Demicco RSS

CD163

p-val:0.891

	Low Risk	Intermediate Risk	High Risk
0	1	0	0
1	12	0	0
2	13	1	0
3	18	1	1

CD68

p-val:0.745

	Low Risk	Intermediate Risk	High Risk
0	25	1	0
1	11	1	1
2	4	0	0
3	4	0	0

PDL-1

p-val:0.522

	Low Risk	Intermediate Risk	High Risk
0	17	0	1
1	18	1	0
2	9	1	0

PD-1

p-val:1.0

	Low Risk	Intermediate Risk	High Risk
0	43	2	1

CD3

p-val:0.291

	Low Risk	Intermediate Risk	High Risk
0	7	0	1
1	23	1	0
2	8	0	0
3	6	1	0

CD8

p-val:0.538

	Low Risk	Intermediate Risk	High Risk
0	16	1	1
1	20	0	0
2	5	1	0
3	3	0	0

CD20

p-val:0.091

	Low Risk	Intermediate Risk	High Risk
0	39	1	1
1	3	0	0
2	1	1	0
3	1	0	0

LAG1

p-val:1.0

	Low Risk	Intermediate Risk	High Risk
0	44	2	1

Sugita RSS

CD163

p-val:0.412

	Low Risk	Intermediate-High Risk
0	1	0
1	11	1
2	14	2
3	12	5

CD68

p-val:0.342

	Low Risk	Intermediate-High Risk
0	21	4
1	9	4
2	5	0
3	3	0

PDL-1

p-val:0.058

	Low Risk	Intermediate-High Risk
0	16	2
1	17	2
2	5	4

PD-1

p-val:1.0

	Low Risk	Intermediate-High Risk
0	37	8

CD3

p-val:0.92

	Low Risk	Intermediate-High Risk
0	6	1
1	19	5
2	8	1
3	5	1

CD8

p-val:0.486

	Low Risk	Intermediate-High Risk
0	13	5
1	17	2
2	6	1
3	2	0

CD20

p-val:0.336

	Low Risk	Intermediate-High Risk
0	35	6
1	1	1
2	1	1
3	1	0

LAG1

p-val:1.0

	Low Risk	Intermediate-High Risk
0	38	8

G-Score RSS

CD163

p-val:0.78

	Low Risk	Intermediate Risk	High Risk
0	1	0	0
1	5	6	1
2	6	9	0
3	9	9	1

CD68

p-val:0.566

	Low Risk	Intermediate Risk	High Risk
0	8	16	2
1	6	5	1
2	3	2	0
3	2	1	0

PDL-1

p-val:0.477

	Low Risk	Intermediate Risk	High Risk
0	6	11	2
1	10	9	0
2	5	4	1

PD-1

p-val:1.0

	Low Risk	Intermediate Risk	High Risk
0	20	24	3

CD3

p-val:0.678

	Low Risk	Intermediate Risk	High Risk
0	3	4	1
1	9	14	1
2	4	4	1
3	5	2	0

CD8

p-val:0.91

	Low Risk	Intermediate Risk	High Risk
0	7	9	1
1	8	11	2
2	4	3	0
3	2	1	0

CD20

p-val:0.234

	Low Risk	Intermediate Risk	High Risk
0	17	23	2
1	1	1	1
2	2	0	0
3	1	0	0

LAG1

p-val:1.0

	Low Risk	Intermediate Risk	High Risk
0	21	24	3

Huang RSS

CD163

p-val:0.314

	Low Risk	High Risk
0	1	0
1	11	1
2	10	3
3	10	6

CD68

p-val:0.312

	Low Risk	High Risk
0	19	4
1	7	5
2	3	1
3	3	0

PDL-1

p-val:0.112

	Low Risk	High Risk
0	15	2
1	13	4
2	4	4

PD-1

p-val:1.0

	Low Risk	High Risk
0	32	9

CD3

p-val:0.378

	Low Risk	High Risk
0	6	1
1	15	7
2	5	2
3	6	0

CD8

p-val:0.78

	Low Risk	High Risk
0	13	4
1	12	5
2	5	1
3	2	0

CD20

p-val:0.71

	Low Risk	High Risk
0	28	10
1	1	0
2	2	0
3	1	0

LAG1

p-val:1.0

	Low Risk	High Risk
0	32	10
