# Supplementary figures and images for: Immunological Tumor Microenvironment of Solitary Fibrous Tumors—Associating Immune Infiltrate with Variables of Prognostic Significance
Source: Cancers (Basel). 2024 Sep 21;16(18):3222. doi: 10.3390/cancers16183222 (PMC11430690; doi:10.3390/cancers16183222)

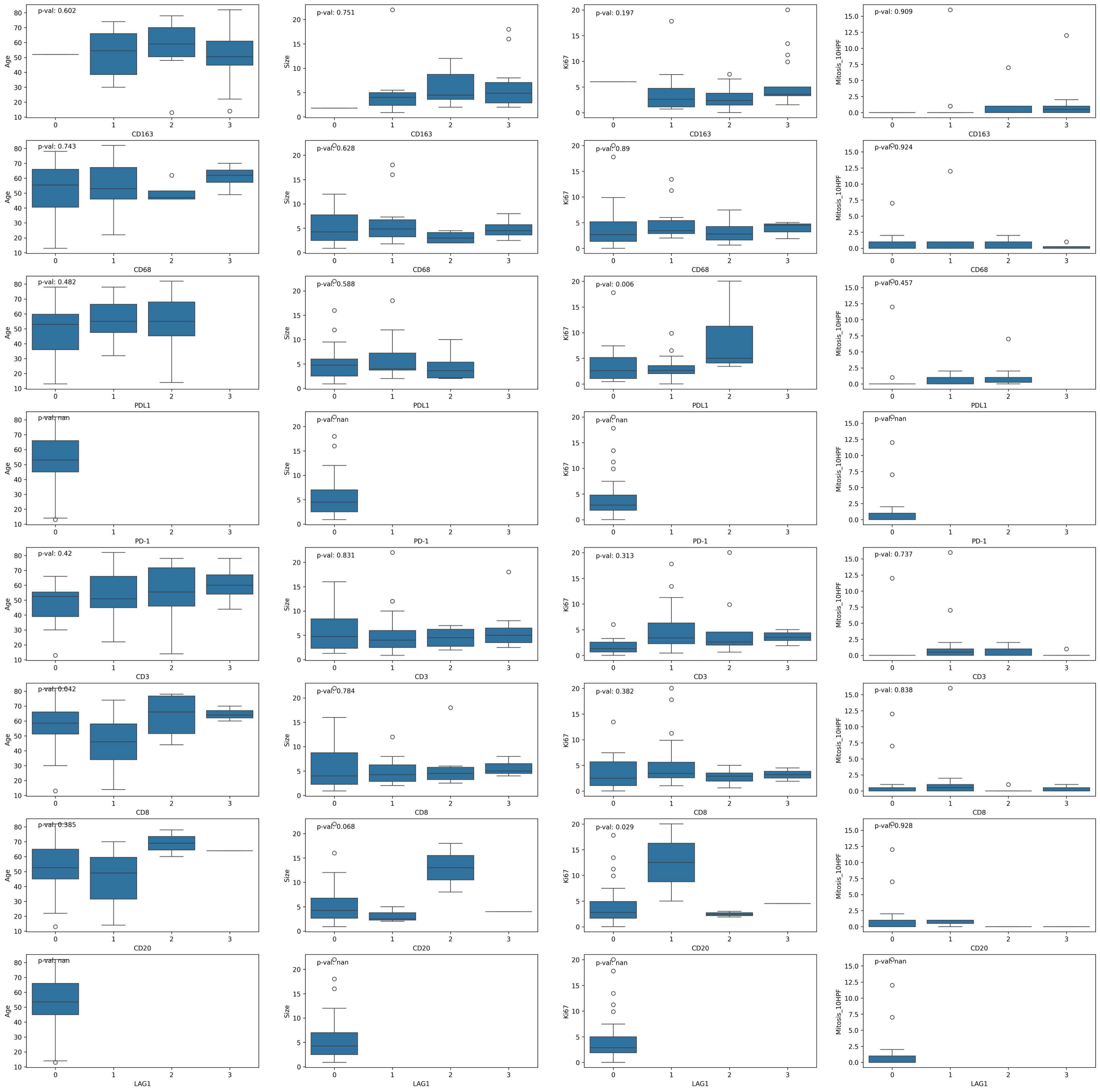

Supplement: Supplementary file 1 [file cancers-16-03222-s001.zip › Sup mat 2.pdf]
